# Filtered Back-Projection Reconstruction with Non-Uniformly Under-Sampled Projections

**Published:** 2022-11-22

**Authors:** Gengsheng L Zeng, Megan Zeng

**Affiliations:** 1 Utah Valley University, Orem, Utah 84058, USA; 2 University of Utah, Salt Lake City, Utah, 84108, USA; 3 University of California at Berkeley, Berkeley, California, 94720, USA

**Keywords:** Data acquisition, Image reconstruction, Signal processing

## Abstract

Tomographic imaging systems normally assume measurements with uniform angular sampling. The view angles are uniformly distributed, and the number of views is approximately the number of the detectors at one view. If the Nyquist sampling criterion is not satisfied, aliasing artifacts may appear in the reconstructed image. If the angular sampling is not uniform, we may be able to reconstruction the image using under-sampled sinograms. This paper presents a case study, which involves a non-uniformly under-sampled sinogram. A closed-form formula is proposed to convert the non-uniformly under-sampled sinogram to uniformly properly sampled sinogram. Finally, the filtered back-projection (FBP) algorithm is used to reconstruct the image. The proposed formula is exact in the sense that the sinogram is band-limited, which is never true in reality.

## Introduction

The Nyquist sampling criterion requires that the sampling rate should be at least twice the bandwidth of the original continuous signal. This criterion assumes the uniform sampling method [1]. If the samples are not uniform, the Lagrange (polynomial) interpolation method has been attempted [2].

Under some conditions, this criterion can be violated. The compress sensing theory is established to recover the original continuous signal by using far fewer samples than required by the Nyquist criterion [3]. In order for the compressed sensing to work, two requirements must be satisfied. The first requirement is the sparsity. The second requirement is incoherence. The sparsity is valid if there is a transform that gives a sparse representation of the original continuous signal. The incoherence is a condition on the eigenvalues of the matrix that is associated with under-determined linear system of the problem. This second requirement is very difficult to verify [4].

In transmission and emission tomography, the patient is scanned with multiple views. The sinogram is discretely sampled. If a tomographic image has a very high resolution, it requires a very small angular sampling interval. Empirically, the required number of views over 180° is N, where N is the number of detection bins to cover the object at one view. The image size is approximately N × N.

In this paper, we do not assume that the continuous sinogram is sparse and is incoherent. We do not require that the sampling in the angular dimension is uniform. This paper uses a toy example and singular value decomposition method to investigate if it is practical to sample the continuous signal by slightly violating the Nyquist sampling criterion. A closed-form formula is derived to convert the non-uniform samples to uniform samples.

## Methods

Here we present a toy example, from which we will gain intuitions on the possibilities of using a sample scheme that violates the Nyquist criterion. Let continuous function g(t) be band-limited; its Nyquist sampling interval is T. In other words, the highest frequency of the continuous signal is 1/ (2T), and the Nyquist sampling rate is 1/T. In tomography, g(t) can be considered as the sinogram for ONE fixed detection bin and t is the view angle. In this application, g(t) is a periodic function with a period of 2p. According to the Nyquist criterion, the continuous function g(t) can be exactly recovered by its samples g(nT). However, g(t) cannot be exactly recovered by the samples g(2nT).

**Figure 1:**
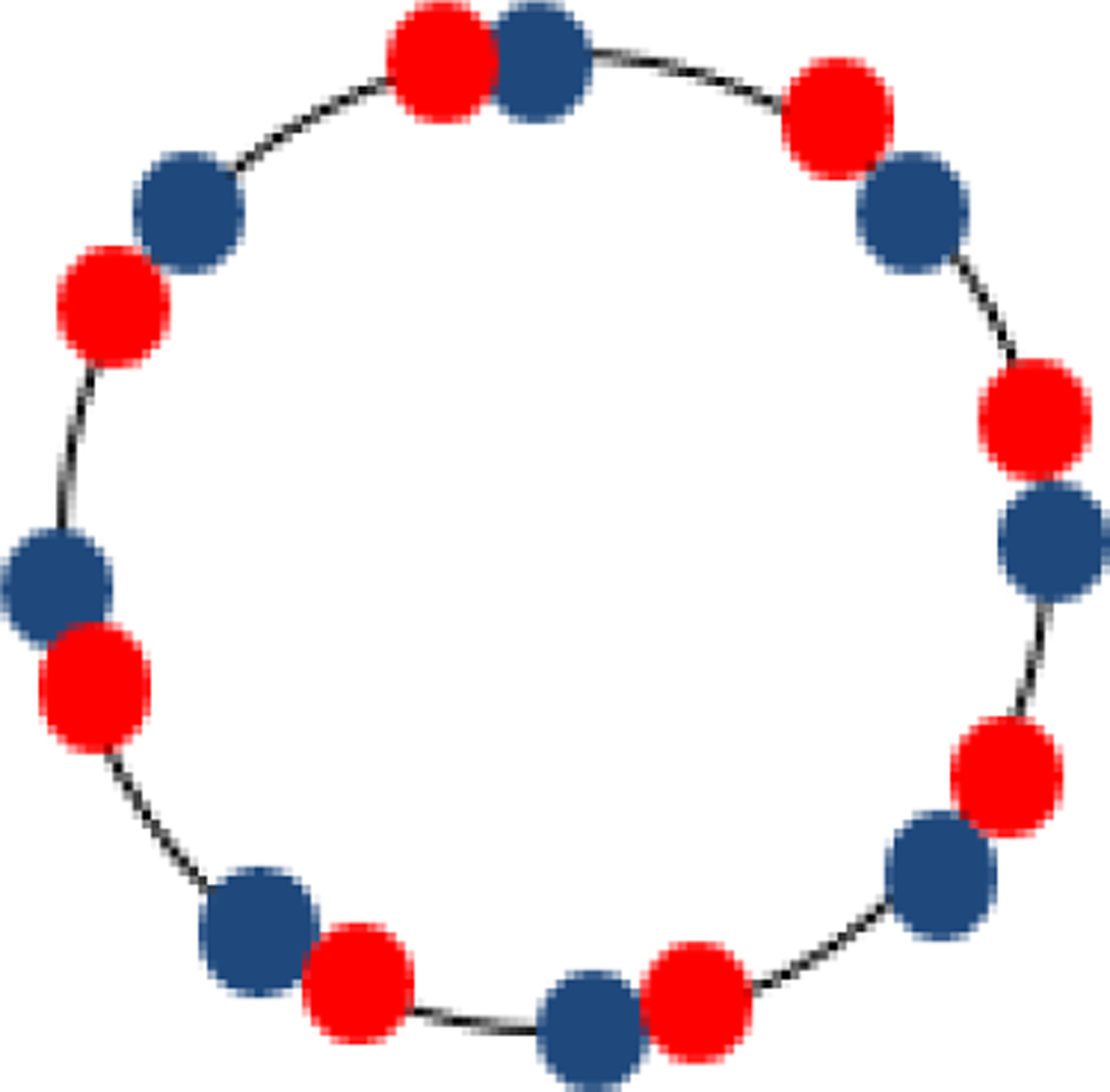
Illustration of two data sets sampled over 360°. The blue dots represent g_1_, and the red dots represent g_2_. The merge of g_1_ and g_2_ gives non-uniform sampling.

Now let us consider a non-uniform sampling situation, which is a combination of two uniformly sampled data sets as illustrated in Fig. 1. Let the discretely sampled signals be

(1) $$g_{1}[n]=g\left(2nT\right)$$

(2) $$g_{2}[n]=g(2nT+\text{Δ}T)$$

where the initial offset DT is set to, for example, T/2. The sampling points have two different gaps between adjacent samples: T/4 or 3T/4. Since 3T/4 > T, the Nyquist sampling criterion is violated (Figure 1).

Question: Can the continuous function g(t) be exactly recovered by the combination of g_1_[n] and g_2_[n]? We will give a YES answer below.

A continuous time-domain impulse train (also known as the Dirac comb function) is defined as

(3) $${\text{Ш}}_{T}(t)=\sum_{k=-\infty }^{\infty }\delta (t-kT),$$

which is a periodic function with a period T. The Dirac comb function can be used to represent the discrete samples. The impulse representations of g_1_[n] and g_2_[n] are

(4) $${\hat{g}}_{1}(t)=g(t){\text{Ш}}_{2T}(t)=\sum_{k=-\infty }^{\infty }g(kT)\delta (t-2kT),$$

(5) $${\hat{g}}_{1}(t)=g(t){\text{Ш}}_{2T}(t)=\sum_{k=-\infty }^{\infty }g(kT)\delta (t-2kT),$$

respectively. Considering the bandwidth of each function and taking the Fourier transform of (4) and (5), in the frequency interval [0, 1/(2T)], we have

(6) $$G_{1}\left(f\right)=\frac{1}{2}G\left(f\right)+\frac{1}{2}G\left(f-\frac{k}{2T}\right),$$

(7) $$G_{1}\left(f\right)=\frac{1}{2}G\left(f\right)+\frac{1}{2}G\left(f-\frac{k}{2T}\right),$$

where G, G_1_, G_2_ are the Fourier transforms of g, g1 and g_2_, respectively. In the matrix form, (6) and (7) become

(8) $$\frac{1}{2}\left[\begin{array}{cc} 1 & 1\\ 1 & e^{\frac{j2\pi \text{Δ}T}{T}} \end{array}\right]\left[\begin{array}{c} G(f)\\ G\left(f-\frac{1}{2T}\right) \end{array}\right]=\left[\begin{array}{l} G_{1}(f)\\ G_{2}(f) \end{array}\right].$$

Solving for G(f) in [0, 1/(2T)],

(9) $$G(f)=\frac{2G_{2}(f)-2e^{j2\pi \frac{\text{Δ}T}{T}}G_{1}(f)}{1-e^{j2\pi \frac{\text{Δ}T}{T}}}.$$

The recoverability of g(t) depends on the ill-condition of the system matrix in (8). If the system (8) is severely ill-conditioned, it is not possible to recover g(t) exactly due to noise.

## Result

**Figure 2:**
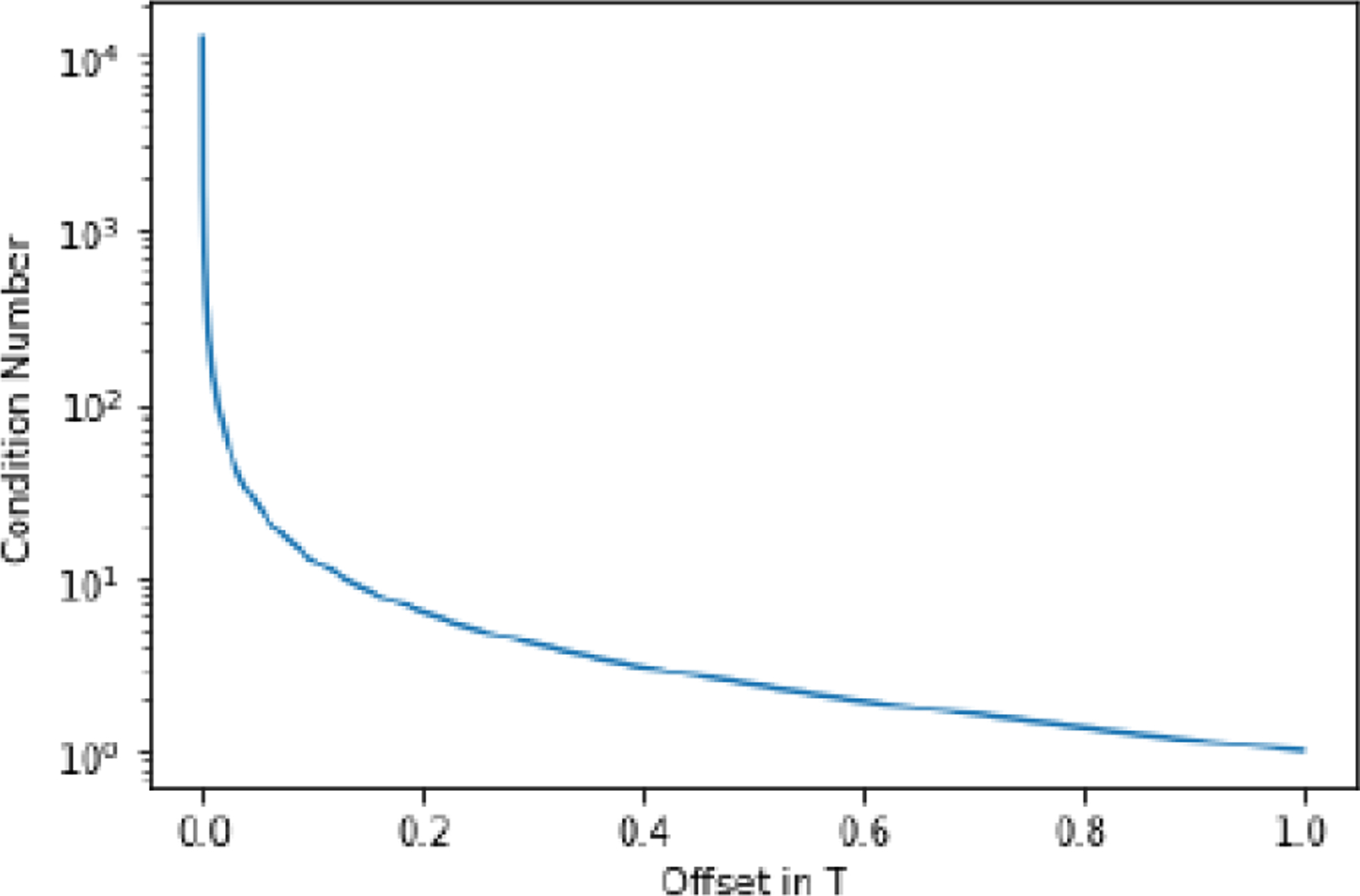
The condition number of the matrix M as a function of ΔT.

Let the 2 × 2 matrix in (8) be denoted as M. The condition number of M is calculated and plotted in Fig. 2. IfΔT=T, this is the uniform sampling situation, and the sampling interval is the Nyquist sampling interval. In this case, the condition number is 1, and the reconstruction is stable. As ΔT tends to 0, the condition number tends to infinity, and the reconstruction becomes too noisy to be useful (Figure 2).

**Figure 3:**
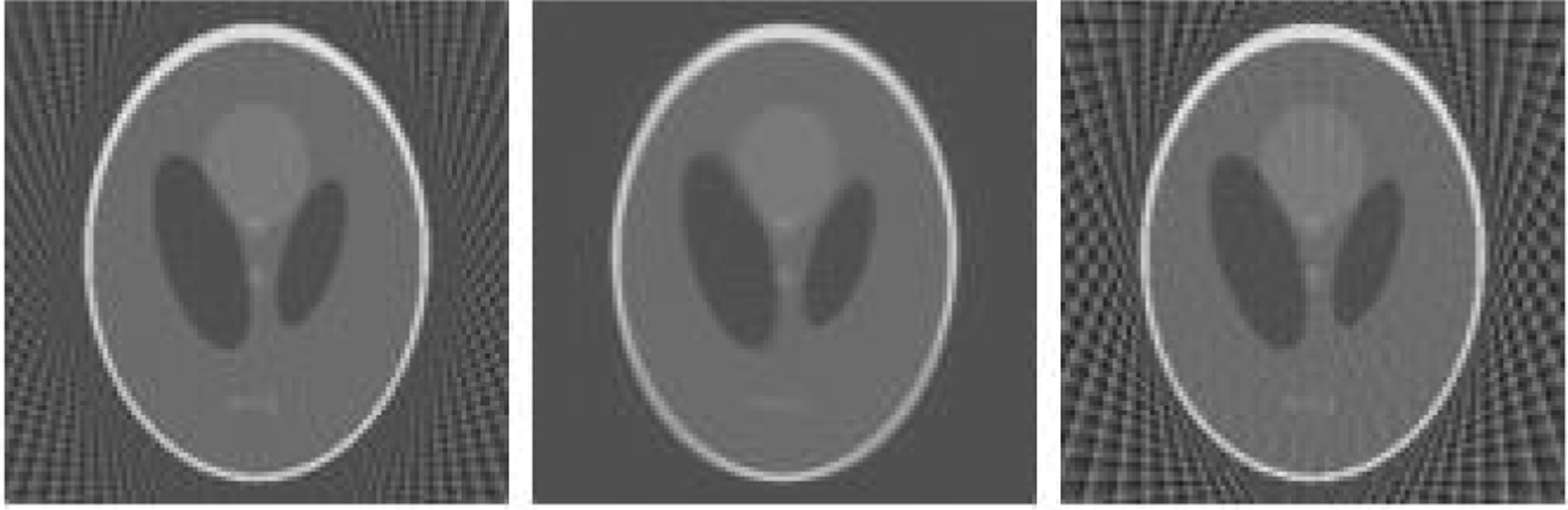
FBP reconstructions. Left: from g1 with angular gap of 8°; Right: from g3 with angular gap of 6°; Middle: from g with proposed formula that combines g_1_ and g_2_.

We finally present a computer simulation of filtered back projection (FBP) reconstruction of the Shepp-Logan phantom, which is a 256 × 256 two-dimensional (2D) image. Two discrete sinograms g_1_ and g_2_ were simulated over 360°, with 8° angular gaps. There were 45 views over 360°. The second set g_2_ had a 2° offset from the first set g_1_. The formula (9) was used to complete the sinogram g from g_1_ and g_2_. The merge of g_1_ and g_2_ had two different angular gaps: 2° and 6°. Another sinogram g_3_ with an angular gap of 6° was simulated. The FBP images from g_1_, g, and g_3_ are shown in Fig. 3 (left), (middle), and (right), respectively. It is observed that in the middle image in the middle of Fig. 3 the angular aliasing artifacts are significantly reduced, but some loss of resolution is also noticed (Figure 3).

## Conclusion

The main result of this paper is the closed-form formula (9), which combines two uniformly sampled measurements and there is a small angular offset ΔT between them. This closed-form formula is derived by assuming the original continuous signal is band-limited with a bandwidth of 1/T. However, this assumption is never met in reality.

After merging the two data sets, the Nyquist sampling criterion is still not satisfied. Due to the non-uniform nature of the merged data set, there is a hope of recovering the original function g(t). This recovering problem is ill-conditioned if the gap ΔT is small.

Our computer simulation uses band unlimited data. Spatial resolution is somewhat degraded by the proposed method. The real-world data are close to band-limited; the resolution degradation issue may not be the same and requires further investigation.
